# Targeting Monocytes and Their Derivatives in Ovarian Cancer: Opportunities for Innovation in Prognosis and Therapy

**DOI:** 10.3390/cancers18020336

**Published:** 2026-01-21

**Authors:** Dharvind Balan, Nirmala Chandralega Kampan, Mohamad Nasir Shafiee, Magdalena Plebanski, Nor Haslinda Abd Aziz

**Affiliations:** 1 Department of Obstetrics and Gynaecology, Faculty of Medicine, National University of Malaysia, Jalan Yaacob Latif, Bandar Tun Razak, Cheras, Kuala Lumpur 56000, Malaysia; dharvind1810@gmail.com (D.B.); nirmala@ppukm.ukm.edu.my (N.C.K.); nasirshafiee@ukm.edu.my (M.N.S.); 2 School of Health and Biomedical Sciences, RMIT University, Bundoora, VIC 3083, Australia; magdalena.plebanski@rmit.edu.au

**Keywords:** ovarian cancer, monocytes, TAMs, DCs, MDSCs, LMR, MLR, prognostic biomarkers

## Abstract

Ovarian cancer remains the most lethal gynaecological malignancy primarily due to late-stage diagnosis, high recurrence rate, and limited treatment efficacy. Current diagnostic tools, including imaging and serum markers, lack sufficient sensitivity and specificity for early detection. Increasing evidence highlights the critical role of myeloid-derived immune cells within the tumour microenvironment in shaping ovarian cancer progression and therapy response. Monocytes and their derivatives are central regulators of immune suppression, chemoresistance, and metastatic dissemination in ovarian tumours. Their recruitment and polarisation are governed by several signalling pathways offering promising therapeutic targets. Strategies including monocyte depletion, TAM reprogramming, MDSC maturation, DC vaccines, and their synergistic use with chemotherapy or immune checkpoint inhibitors are being explored to restore anti-tumour immunity in ovarian cancer. Parallel to therapeutic potential, the lymphocyte-to-monocyte ratio and its reciprocal monocyte-to-lymphocyte ratio have also emerged as potential accessible and cost-effective prognostic tools that predict disease aggressiveness and survival in ovarian cancer. This review features the diagnostic, prognostic, and therapeutic significance of monocytes and their derivatives in ovarian cancer management and highlighting new opportunities for next-generation immunomodulatory therapies.

## Simple Summary

Ovarian cancer is a serious disease that is often discovered at an advanced stage with limited treatment options and modest outcomes. This review explores the role of immune cells, mainly monocytes and their derivatives, which can either fight cancer or help it grow. This review also highlights how these cells contribute to ovarian cancer development and how they might be targeted to improve treatment outcomes. We also discuss the imbalance between different immune cells, and this may help predict the disease progression and patient outcomes. By understanding how monocytes and their related cells influence ovarian cancer, researchers and clinicians may be able to design better diagnostic tools and more effective personalised therapies in the future.

## 1. Introduction

Ovarian cancer is one of the most lethal gynaecological malignancies, frequently diagnosed at an advanced stage due to its non-specific early symptoms. Globally, more than 324,000 new cases and over 200,000 deaths are reported annually, with survival rates varying significantly by stage at diagnosis [1]. While patients with early-stage disease (stage I–II) may achieve 5-year survival rates of 70–95%, those with advanced-stage disease (stage III–IV) typically have survival rates of only 10–40%, despite undergoing an aggressive treatment regimen [2]. Current diagnostic strategies include clinical assessments, imaging modalities, and serum markers, including cancer antigen 125 (CA-125) and human epididymis protein 4 (HE4); however, early detection remains hindered by the poor sensitivity and specificity of existing biomarkers [3]. Standard treatment consists of cytoreductive surgery followed by platinum-based chemotherapy and offers limited long-term benefit, while immunotherapy has thus far shown modest efficacy [4]. Nevertheless, most patients with advanced ovarian cancer experience relapse within two years [5,6]. These realities underscore the need for new prognostic markers and innovative therapeutic strategies that target the underlying biology of ovarian cancer.

Immune components of the tumour microenvironment (TME) are increasingly recognised as critical determinants of ovarian cancer progression immune escape, chemoresistance, and clinical outcome. Monocytes, macrophages, dendritic cells (DCs), and myeloid-derived suppressor cells (MDSCs) represent a diverse compartment of myeloid mononuclear cells (MMCs) that function at the intersection of innate and adaptive immunity. Traditionally considered a homogeneous population, recent advances such as single-cell RNA sequencing and lineage-tracing studies have demonstrated their remarkable heterogeneity, shaped by distinct ontogenetic pathways, tissue-specific conditioning, and microenvironmental signals [7,8]. These cells not only mediate pathogen sensing and antigen presentation but also exert regulatory and effector roles in chronic inflammation and tissue remodelling, positioning them as central players in cancer biology.

Monocytes and their downstream derivatives form a highly dynamic myeloid network within the ovarian tumour microenvironment, collectively shaping tumour behaviour through immunosuppression, angiogenesis, and modulation of anti-tumour immunity. Circulating monocytes are actively recruited into the TME, where they differentiate predominantly into tumour-associated macrophages (TAMs), the most abundant myeloid subset in ovarian cancer [9]. These TAMs typically polarise toward an M2-like phenotype characterised by pro-angiogenic, pro-survival, and immunosuppressive functions [9]. M2-like TAMs activate nuclear factor kappa-light-chain-enhancer of activated B cells (NF-κB) and signal transducer and activator of transcription (STAT) 3 signalling in cancer cells, and a high M2/M1 ratio is consistently associated with poor prognosis [9,10,11,12]. Beyond TAMs, monocyte-derived DCs often acquire tolerogenic or dysfunctional phenotypes in the ovarian cancer milieu, impairing antigen presentation and limiting effective T-cell priming, thereby enabling tumour immune evasion. In parallel, monocytic myeloid-derived suppressor cells (M-MDSCs) expand markedly in ovarian cancer and exert potent suppression of T-cell and NK-cell activity through arginase-1 (ARG1) activity, nitric oxide production, and secretion of immunosuppressive cytokines. Together, these monocyte-lineage populations secrete a wide range of immunomodulatory and angiogenic factors including vascular endothelial growth factor (VEGF), platelet-derived growth factor (PDGF), interleukin (IL)-10, and transforming growth factor beta (TGF-β) that enhance neovascularisation, restrict cytotoxic immunity, facilitate metastatic dissemination, and contribute to chemotherapy resistance [13,14,15]. Collectively, monocytes, TAMs, DCs, and M-MDSCs form an interdependent immunosuppressive network that drives ovarian cancer progression and represents a compelling target for therapeutic intervention.

The pro-tumourigenic role of monocytes and their differentiation into TAMs within the ovarian TME has made them an important therapeutic target. Current strategies include TAM depletion, reprogramming their M2-like phenotype, and combining these tactics with conventional therapies to enhance treatment response and to overcome chemotherapy resistance. Growing interest in monocyte-driven pathology has expanded therapeutic strategies beyond TAM-focused interventions to encompass the broader myeloid compartment, including monocytes, DCs, and MDSCs. Targeting monocyte recruitment and differentiation represents a foundational approach, with inhibition of the colony-stimulating factor 1/colony-stimulating factor 1 receptor (CSF-1/CSF-1R) axis shown to reduce monocyte influx into the tumour bed and limit their maturation into pro-tumourigenic TAMs [16]. Within the macrophage lineage, therapies aim to deplete TAMs, block their M2-polarising signals, or reprogramme them toward an anti-tumour M1 phenotype using immunomodulators, nanocarriers, or pathway-specific inhibitors, leading to reduced tumour burden and restored chemosensitivity [17]. Parallel efforts to modulate monocyte-derived DCs seek to overcome their tumour-induced tolerogenic state; strategies include enhancing DC activation, restoring antigen presentation capacity, and promoting effective T-cell priming to counteract ovarian cancer’s immune-evasive TME. MDSCs are also therapeutically actionable along four major avenues: depleting MDSCs, inhibiting their suppressive mechanisms, blocking their recruitment, and promoting their differentiation into non-suppressive myeloid lineages. Together, these diversified strategies highlight the therapeutic potential of targeting monocytes, macrophages, DCs, and MDSCs to reshape the myeloid landscape and improve outcomes in ovarian cancer.

Researchers have increasingly focused on accessible, non-invasive inflammatory biomarkers in blood, like the lymphocyte-to-monocyte ratio (LMR) and its reciprocal, the monocyte-to-lymphocyte ratio (MLR), because traditional prognostic markers like residual tumour size and platinum sensitivity are limited to postoperative assessments [18,19]. These ratios reflect the balance between anti-tumour lymphocytes and pro-tumour monocytes, with a high LMR or low MLR indicating a favourable prognosis [20]. However, despite variation in cutoff values across studies, the prognostic trend remains consistent, whereby a low LMR or high MLR is associated with poor survival [21]. Biologically, lymphocytes drive cytotoxic responses, while monocytes differentiate into TAMs that promote angiogenesis, metastasis, and immunosuppression [22]. Clinical studies and meta-analyses consistently confirm a low LMR as an independent predictor of poor overall survival (OS) and progression-free survival (PFS) in epithelial ovarian cancer (EOC) and other malignancies [23,24]. Furthermore, a low LMR correlates with aggressive disease features, including advanced International Federation of Gynaecology and Obstetrics (FIGO) stage, poor differentiation, ascites, and high CA-125 levels [25,26]. Despite limitations such as inconsistent cutoffs and reliance on retrospective cohorts, LMR/MLR remains a promising, cost-effective prognostic tool justifying prospective clinical validation. Given the central role of monocytes and their derivatives in ovarian cancer biology, prognosis, and therapeutic response, this review synthesises current evidence on their diverse functions and evaluates emerging diagnostic, prognostic, and therapeutic strategies targeting this deadly gynaecological malignancy.

## 2. Monocytes, Macrophages, Dendritic Cells, and Myeloid-Derived Suppressor Cells: Origins, Functions, and Evolving Concepts in Ovarian Cancer

**Figure 1:**
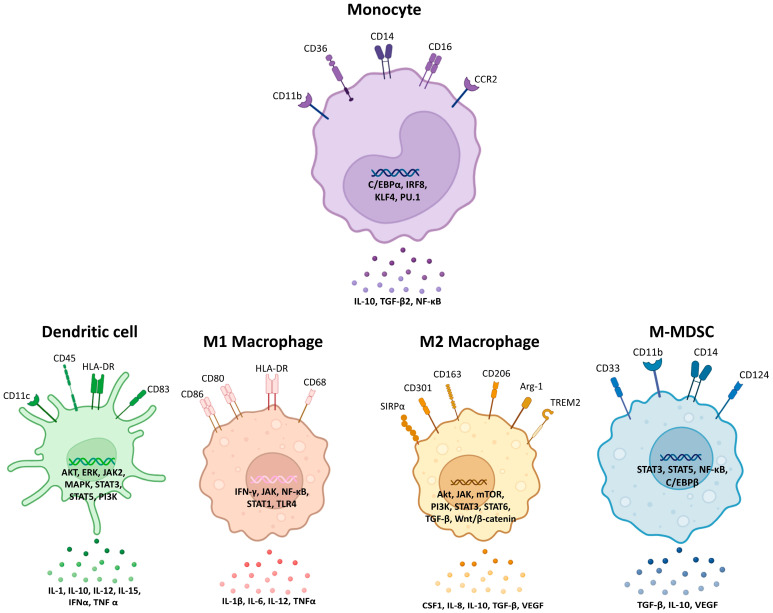
Key cell surface markers, signalling pathways, and secreted cytokines of monocytes, macrophages, dendritic cells, and myeloid-derived suppressor cells (created with BioRender.com).

Monocytes, macrophages, DCs, and MDSCs constitute the MMC population, a central component of the immune system that integrates innate immune surveillance with the initiation and regulation of adaptive immune responses. These cells are equipped with sophisticated sensing mechanisms that enable the detection and endocytosis of pathogens and cellular debris, while their antigen-presenting capacity allows them to serve as a critical bridge between the innate and adaptive immunity [27]. In addition to their sentinel functions, MMCs contribute substantially to immune homeostasis by mediating effector processes such as the resolution of inflammation, clearance of apoptotic cells, and facilitation of tissue repair and regeneration [28]. Distinct transcriptional programmes, cell surface marker expression profiles, and lineage-determining signalling pathways have been shown to govern their specialisation, while cytokine-driven signalling within the tissue microenvironment further shapes their phenotypic and functional heterogeneity [29]. These emerging insights highlight that MMCs do not represent a static or uniform system, but rather a dynamic and highly adaptable network of cell populations whose functions are tailored by developmental origins and local environmental contexts. Ovarian cancer progression is profoundly influenced by diverse myeloid populations that collectively shape the TME and systemic immune landscape. As summarised in Figure 1, the differentiation trajectories of monocytes, macrophages, DCs, and MDSCs are orchestrated by a combination of intrinsic molecular regulators and extrinsic cytokine-mediated signals, which together ensure their ability to perform diverse and context-dependent roles in recruitment, differentiation, immunosuppression, chemoresistance, and metastasis. These cells, while critical for normal host defence, tissue homeostasis, and immune surveillance, are dynamically reprogrammed within ovarian tumours to support tumour growth, immune evasion, chemoresistance, and metastatic dissemination. In this section, we examine each cell type, highlighting their physiological functions, recruitment and differentiation in the tumour context, and their contributions to immune evasion, therapy resistance, and metastasis. Table 1 provides a concise overview of the mechanisms by which key myeloid populations drive immunosuppression, chemoresistance, and metastasis in ovarian cancer. Understanding these dynamic roles provides insights into potential biomarkers and therapeutic targets that could reshape ovarian cancer management.

### 2.1. Monocytes

Monocytes represent the major circulating subset of human myeloid cells and comprise approximately 10% of peripheral leukocytes [30]. Beyond their classical roles in inflammation and host defence, monocytes are now recognised as a heterogeneous and highly complex population capable of rapidly differentiating into macrophages, dendritic cells, or M-MDSCs in response to tissue-specific signals [31]. Monocytes arise from common monocyte progenitors (cMoPs) in bone marrow, characterised by CD117, CD135, CLEC12A, and CD64 expression [32]. Their development is tightly controlled by transcription factors such as PU.1, C/EBPα, IRF8, and KLF4, which regulate monocyte identity, inflammatory responses, and differentiation potential [33,34,35,36]. Human monocytes are broadly classified into three subsets based on differential CD14/CD16 expression, which are classical (CD14^+^CD16^−^), intermediate (CD14^high^CD16^+^), and non-classical (CD14^low^CD16^+^), each exhibiting distinct inflammatory, antigen-presenting, and migratory properties [37,38]. The heterogeneity of circulating monocytes is critical in ovarian cancer, where monocyte-derived cells are among the earliest and most abundant immune infiltrates recruited to the TME, shaping tumour progression, immune suppression, and therapeutic resistance [39,40]. Their recruitment, differentiation, and phenotypic adaptation are increasingly recognised as central drivers of ovarian cancer progression. The circulating monocytes, which are recruited to ovarian tumours, undergo rapid reprogramming toward pro-tumourigenic states under the influence of TME-derived cytokines, metabolic cues, and stromal interactions. Ovarian cancer cells and omental stromal tissue secrete chemokines such as CCL2 and CXCL12, which promote monocyte trafficking into metastatic niches, particularly the omentum [9]. Once recruited, these monocytes differentiate predominantly into M2-like TAMs that support tumour invasion, angiogenesis, and immune evasion [41,42].

In the majority population, classical monocytes are the primary reservoir for TAMs. These cells express high levels of the chemokine receptor CCR2, facilitating their rapid and abundant recruitment into the tumour and peritoneal ascites in response to TME-derived chemokines like CCL2, where they subsequently differentiate into immunosuppressive, pro-angiogenic M2-like TAMs [43]. Meanwhile, the intermediate monocyte subset shows the most robust expansion in the peripheral blood of ovarian cancer patients, and their increased frequency is positively associated with peritoneal tumour burden, suggesting they are a crucial circulating biomarker of disease progression. These intermediate monocytes exhibit a highly inflammatory phenotype and are specifically linked to the lower effector/regulatory T-cell ratio within the ascites, thereby promoting systemic and local immune suppression [43]. Recent single-cell RNA sequencing studies reveal that these subsets are further diversified in ovarian cancer, where transcriptionally distinct monocyte clusters emerge upon exposure to tumour-derived factors [44,45]. In high-grade serous ovarian cancer, intermediate monocytes expand in the peripheral blood and showed elevated expression of HLA-DR, CCR2, CD36, and inflammatory chemokine receptors, suggesting a primed state biassed toward differentiation into TAMs once they enter the TME [44]. Finally, non-classical monocytes act as vascular sentinels, patrolling the endothelium and contributing to the overall inflammatory milieu by secreting pro-inflammatory cytokines, which indirectly supports the chronic inflammation necessary for tumour survival and metastatic spread [46].

In addition to serving as precursors to TAMs, monocytes in ovarian cancer patients also function as dynamic systemic immune effectors whose altered phenotype and frequency and potentially influence the disease status. A study of chemotherapy-naïve ovarian cancer patients (NCT02978755) demonstrated a significant expansion of circulating intermediate monocytes compared with healthy controls. The ovarian cancer patients also showed immunosuppressive ascites features, elevated tumour burden, and reduced effector/regulatory T-cell ratios in the peritoneal fluid, suggesting that circulating monocytes may contribute to the immunological landscape of the TME [43]. Moreover, monocytes isolated from patient ascites were shown to actively produce immunoregulatory cytokines such as IL-10 and TGF-β2, suppressing the proliferation and cytokine production of autologous T cells, therefore being directly involved in promoting local immune tolerance [47]. In addition, Zheng et al. (2023) showed that ovarian cancer ascites contain a distinct and diverse immune ecosystem, including specialised monocyte/macrophage subsets not seen in solid tumour tissue [48]. Their single-cell analysis identified tumour-enriched macrophages derived from circulating monocytes and ascites-enriched macrophages with tissue-resident, embryonic-origin signatures, revealing dual ontogenies within the ascites’ compartment. These findings demonstrate that both monocyte-derived and non-monocyte-derived macrophage populations shape the ascites’ immune niche, with implications for metastasis, immune regulation, and therapeutic responsiveness. Additionally, monocyte-derived ascites-conditioned macrophages secrete soluble mediators, transforming growth factor beta-induced protein, tenascin C, and fibronectin that remodel extracellular matrix and support tumour cell adhesion, migration, and metastasis, further highlighting that monocyte to macrophage transition is a continuum that begins within the circulating monocyte pool and contributes to ovarian cancer progression from early stages [49].

Monocytes play a critical role in the progression of ovarian cancer by actively orchestrating both chemoresistance and metastatic dissemination upon their recruitment to the TME. Driven by chemokines such as CCL2, monocytes infiltrate the tumour and differentiate into TAMs or MDSCs, which constitute a significant portion of the immune infiltrate. Recent research demonstrates that platinum-based chemotherapy can paradoxically enrich specific pro-inflammatory macrophage phenotypes that secrete cytokines like CCL2, directly supporting the maintenance and expansion of aldehyde dehydrogenase-positive (ALDH+) ovarian cancer stem-like cells (CSCs), thereby facilitating disease recurrence [50]. Furthermore, TAMs have been shown to modulate the DNA damage response in OC cells; specifically, they upregulate polymerase ŋ-mediated translesion DNA synthesis, allowing cancer cells to bypass cisplatin-induced DNA adducts and survive cytotoxic treatment [51]. In the context of metastasis, monocytes and tissue-resident macrophages are pivotal in preparing the “pre-metastatic niche,” particularly within the omentum. CD163^+^ Tim4^+^ omental macrophages have been identified as key drivers of metastatic spread, fostering an environment that supports the initial seeding and colonisation of circulating tumour cells [9]. Additionally, exosomes derived from CD163^+^ TAMs transfer microRNAs, such as miR-221-3p, to OC cells, which activate the AKT signalling pathway and promote epithelial–mesenchymal transition (EMT), further enhancing both metastatic potential and drug resistance [52]. Etzerodt et al. demonstrated that omentum-resident macrophages expand during metastatic colonisation and actively recruit circulating monocytes, which subsequently adopt pro-tumour functions including extracellular matrix (ECM) remodelling and support of cancer cell invasion [9]. Additional mechanistic insight comes from ovarian cancer-derived extracellular vesicles (EVs), which deliver lipids, microRNAs, and immunomodulatory molecules that directly instruct monocytes to assume M2-like phenotypes. Tang et al. showed that ovarian cancer EVs enhanced STAT3 activation and induced secretion of IL-10, VEGF, and matrix-remodelling enzymes in monocyte-derived macrophages, thereby promoting angiogenesis and tumour progression [41]. Other tumour-derived secreted proteins, such as periostin, drive integrin-mediated activation of NF-κB and TGF-β2 signalling in monocytes, heightening their differentiation into TAMs and supporting metastatic dissemination [42].

Collectively, these findings support monocytes in ovarian cancer as both sentinels of systemic tumour-induced immune dysregulation and as active participants in immunosuppression, tumour cell support, and microenvironment remodelling, before and beyond their differentiation into classical TAMs. Together, these mechanistic insights emphasise that monocytes are not passive precursors but active, plastic contributors to tumour progression. Their dynamic reprogramming within the ovarian TME underscores their potential both as clinical biomarkers and as therapeutic targets for strategies aiming to recondition the TME or prevent immunosuppressive macrophage accumulation.

### 2.2. Macrophages

Macrophages are a key component of the innate immune system, known for their adaptability and multifunctionality. They serve critical roles in host defence, immunological regulation, tissue remodelling, and homeostasis [53]. Macrophages in various organs polarise in response to changes in their environment, creating distinct macrophage subtypes such as classically activated macrophages, M1 macrophages, and alternatively activated macrophages, M2 macrophages [54]. M2 macrophages are further clustered into four distinct subtypes (M2a, M2b, M2c, and M2d) based on the activating stimuli they encounter and their resulting specific anti-inflammatory and tissue-remodelling functions. The four M2 macrophage subtypes primarily function in tissue repair and fibrosis (M2a), immune regulation (M2b), immunosuppression and efferocytosis (M2c), and angiogenesis and tumour promotion (M2d) [55]. The microbial component LPS can induce interferon-gamma (IFN-γ) and promote macrophage polarisation to the M1 phenotype, whereas IL-4, IL-13, or IL-10, which are associated with tissue repair and remodelling, can induce macrophage differentiation to the M2 phenotype [56]. M1 macrophages are pro-inflammatory, producing cytokines like TNF-α, IL-1β, IL-6, and IL-12 for host defence against pathogens, while M2 macrophages produce anti-inflammatory cytokines like IL-8, IL-10, TGF-β, and CSF1 for wound healing, tissue remodelling, and fibrosis [57]. M1 macrophages express high levels of surface markers like MHC class II, CD68, and costimulatory molecules (CD80 and CD86), which are associated with pro-inflammatory cytokines secretion, antigen presentation, and T-cell activation [58]. M2 macrophages, on the other hand, express high levels of surface markers like CD163, ARG1, mannose receptor (CD206), and CD301, associated with tissue repair and immunosuppression [59,60].

Macrophage polarisation is the process by which macrophages, which are adaptable immune cells, switch between functional states in response to signals from their environment. They can either polarise into a pro-inflammatory (M1) state, which combats infections and promotes anti-tumour activity, or an anti-inflammatory (M2) state, which promotes tissue healing but can also facilitate tumour development and metastases [61]. This functional switch allows them to influence key pathological processes, including angiogenesis, metastasis, and chemotherapy resistance in ovarian cancer [62]. The ovarian TME coordinates the recruitment of monocytes from the peripheral circulation. Tumour cells and associated stromal cells produce chemokines and cytokines, such as CCL2 and CSF-1, which effectively recruit these immune cells to the ovarian TME [63]. This mechanism triggers invading monocytes to differentiate into TAMs, and subsequently, local stimuli inside the TME drive these TAMs to a pro-tumourigenic, M2-like phenotype [64]. M2 polarisation, which is closely associated with tumour promotion, is stimulated by IL-4, IL-10, and TGF-β, largely through the JAK/STAT6, PI3K/AKT/mTOR, and TGF-β/SMAD pathways, and promotes tissue repair, immune suppression, and angiogenesis [65]. M2-like TAMs suppress cytotoxic T-cell and NK-cell activity through the secretion of IL-10 and TGF-β, the upregulation of checkpoint molecules such as programmed death-ligand 1 (PD-L1), and the metabolic depletion of nutrients required for T-cell activation [66]. Evidence suggests that M2-like phenotype promotes ovarian cancer progression through specific mechanisms, including the secretion of pro-survival factors that activate pathways such as NF-κB and STAT3 within cancer cells [62]. Furthermore, a high ratio of M2-like to M1-like macrophages is often linked with a poor prognosis, while the vice versa is associated with improved survival in ovarian cancer patients [67]. High-grade serous ovarian cancer patients who have a higher ratio of pro-inflammatory M1 macrophages to immunosuppressive M2 macrophages, leading to longer OS, PFS, and platinum-free interval regardless of surgery extent, indicating TAM polarisation’s importance in patient outcomes. Moreover, patients with platinum-sensitive malignancies had a larger M1/M2 ratio, indicating that M1 polarisation also improves treatment efficiency [68]. On the other hand, chemotherapy can reshape macrophage polarisation and phenotype via altering myeloid-cell networks within the tumour microenvironment. In a recent study, the authors analysed paired primary and recurrent ovarian cancers, revealing that recurrent, platinum-based chemotherapy-exposed tumours bearing wild-type BRCA lost prior TIL:DC immune niches and instead accumulated immunosuppressive myeloid landscapes dominated by TREM2/ApoE-high TAMs [69]. This shift suggests chemotherapy contributes to phenotype remodelling of macrophages toward suppressive TAM states, altering the balance of immune-regulatory myeloid cells and potentially facilitating immune evasion.

Following differentiation, TAMs are playing a central role in establishing an immunosuppressive microenvironment within the TME. The M2-like phenotype is characterised by the elevated secretion of anti-inflammatory cytokines, including IL-10 and TGF-β. In the immunosuppressive microenvironment of ovarian tumours, signals such as IL-10 and TGF-β drive macrophages toward M2 polarisation via pathways including PI3K/AKT/mTOR and TGF-β/SMAD, endowing them with immune-regulatory, tissue-remodelling, and pro-tumour functions [70]. These M2-like TAMs, characterised by high expression of markers such as CD163, CD206, ARG1, SIRPα, and TREM2 and elevated production of IL-10 and TGF-β, create a suppressive milieu that blunts effective anti-tumour immunity, supports angiogenesis and extracellular matrix remodelling, and facilitates cancer cell survival, invasion, and metastasis [59,60,71,72]. This cytokine profile effectively suppresses the activity of primary anti-cancer immune cells, such as cytotoxic T-lymphocytes and NK cells, which are responsible for identifying and destroying tumour cells. TAMs directly inhibit the proliferation and function of effector immune cells by secreting these cytokines, which attract and activate Tregs, which further intensifies the immunosuppressive environment surrounding the tumour [70]. This immune-privileged sanctuary protects the tumour from immune surveillance and elimination, which then leads to poor prognosis. In ovarian cancer patients, the upregulation of IL-10 and TGF-β contributes to immune evasion and correlates with a poorer OS [73]. Multiple studies have confirmed that high levels of IL-10 and TGF-β in the serum, ascites, and tumour tissue of ovarian cancer patients are significantly associated with advanced disease stage, shorter PFS, and worse OS [74,75]. Moreover, the dominance of IL-10/TGF-β–producing TAMs is associated with therapy resistance: in platinum- or chemotherapy-treated ovarian cancers, increased M2/TAM infiltration (and the associated immunosuppressive cytokine milieu) correlates with reduced chemosensitivity and poorer clinical outcomes.

In addition, the presence of TAMs strongly influences the development of resistance to chemotherapy, a major clinical challenge in the management of ovarian cancer. The intricate cellular crosstalk between TAMs and cancer cells, often mediated by secreted factors and direct cell-to-cell contact, can activate pro-survival signalling pathways such as NF-κB and STAT3 within the tumour cells [76]. This renders the cancer cells less susceptible to apoptosis induced by chemotherapeutic agents. Therefore, the protective function of TAMs undermines the efficacy of standard platinum-based treatments and is a significant factor in the frequent recurrence and poor prognosis observed in patients with advanced disease [77]. Clinically, the presence of a high density of M2-like TAMs is often associated with shorter PFS and OS in patients with high-grade serous ovarian cancer [78,79]. The development of platinum resistance, often mediated by these TAMs, is a critical prognostic factor, with patients experiencing disease recurrence within six months of first-line therapy having a median PFS of just 9–12 months [70,78].

Macrophages also play a critical role in facilitating metastasis, i.e., the dissemination of cancer cells to distant sites. M2-like TAMs secrete matrix metalloproteinases (MMPs), which degrade the protein components of the extracellular matrix that are vital for tissue remodelling, thus creating physical pathways for ovarian cancer cells to invade the surrounding stroma and enter blood or lymphatic vessels [25]. In ovarian cancer, this often leads to metastasis within the peritoneal cavity, including the omentum and diaphragm, as well as to more distant sites like the liver, lungs, and bone [80]. In addition to enabling migration, TAMs can also serve as co-travellers, migrating alongside tumour cells and assisting their survival during transit through circulation. They can then aid in the migration of tumour cells at secondary sites, helping them to colonise and establish new metastases [62]. A pre-clinical study led by Krishnan V demonstrates that omental tissue-resident macrophages secrete high levels of CCL6/CCL23, which attract ovarian cancer cells and significantly enhance their migration [81]. These chemokines activate CCR1 on tumour cells, triggering ERK1/2 and PI3K/AKT signalling that drives cytoskeletal reorganisation and motility. Blocking CCR1 effectively suppresses omental colonisation, establishing the CCL6/CCL23–CCR1 axis as a key driver of early metastatic seeding in ovarian cancer. In a recent in vivo study, the authors found that macrophage-secreted CCL9/CCL5 induces CCR1-mediated ovarian cancer metastasis to the omentum in the absence of CCL6 [82]. The study showed that, in the absence of omental ligand CCL6, CCL9/CCL5 were sufficient to drive ovarian cancer cell homing and colonisation in the peritoneal cavity. Blocking CCR1 significantly reduced omental metastasis, indicating that the CCL9/CCL5–CCR1 axis is functionally necessary for this process. Through these data, the authors define a novel paracrine chemokine signalling loop derived from macrophages that support tissue-specific metastasis of ovarian cancer, pointing to macrophage-secreted CCL9 and CCL5 as key modulators of the tumour microenvironment and cancer dissemination. On the other hand, clinical studies demonstrate that a high density of M2-like TAMs (CD163^+^) in the omentum is an independent adverse prognostic factor for OS in patients with advanced EOC. One retrospective study (*n* = 110) found that elevated CD163^+^ cell counts and a high CD163/CD68 ratio both independently predicted poorer PFS and OS [83]. This observation is reinforced by a meta-analysis encompassing 794 ovarian cancer patients showed that high CD163^+^ TAM density was linked to significantly worse PFS, and that a lower M1/M2 TAM ratio predicted poorer OS outcomes [84].

In conclusion, the pro-tumourigenic functions of M2-like TAMs are a central mechanism driving ovarian cancer progression, metastasis, immune suppression, and chemoresistance. The presence of a high density of these cells is a robust indicator of poor prognosis and reduced survival rates in this gynaecological cancer patients. Therefore, a promising way forward for managing ovarian cancer involves developing therapeutic strategies that specifically target TAMs. TAMs can be targeted by limiting their recruitment, reprogramming them to an anti-cancer M1-like phenotype, or restoring their capacity to phagocytose tumour cells, all of which can help reverse immunosuppression and increase immunotherapy efficacy. By addressing these pro-tumourigenic functions, we may be able to significantly improve the efficacy of existing treatments and ultimately enhance long-term ovarian cancer patient survival.

### 2.3. Dendritic Cells

Dendritic cells are professional antigen-presenting cells (APCs) that play a critical role in the initiation and modulation of innate and adaptive immune responses. Their strategic position at body surfaces, functional flexibility, and capacity to stimulate naïve T cells make them essential in immunological surveillance and host defence. They live in lymphoid tissues as resident populations, whereas migratory DCs go to draining lymph nodes to initiate and regulate adaptive immune responses [85]. DCs have specific cell-surface markers such as HLA-DR, CD83, CD11c, and CD45 that have been widely utilised to characterise them, in addition to their distinctive functional features [86,87]. Dendritic cells develop from bone marrow stem cells via a common dendritic cell progenitor, resulting in two major DC lineages. Human conventional dendritic cells (cDCs) are extremely unique and often seen as granulocytes or monocytes, based on the expression of antigens such as CD13, CD33, CD11b, and CD11c [88]. cDCs consist of two major subsets: cDC1 (CD141^+^) and cDC2 (CD1c^+^). cDC1s are specialised in the cross-presentation of antigens to CD8^+^ T cells, while cDC2s primarily activate CD4^+^ T cells and modulate helper T-cell production [89]. Plasmacytoid DCs (pDCs) characterised by CD123^+^ expression are potent producers of type I interferons (IFN-α/β) and are key players in antimicrobial defence [90]. pDCs are conserved in numerous mammalian species, including humans, where they express CD123, CD303, and CD304 as cell surface markers [91].

Dendritic cells are central orchestrators of anti-tumour immunity, yet in ovarian cancer, their functions are greatly dysregulated within both the TME and malignant ascites. Studies have demonstrated that malignant ascites significantly impair DC maturation and T-cell stimulation capacity, further underscoring the immunosuppressive potential of DCs in ovarian cancer. An earlier study by Brencicova et al. showed ascites from ovarian carcinoma patients markedly reduces Toll-like-receptor-mediated upregulation of activation marker CD86 on monocyte-derived DCs and suppresses their production of IL-12, IL-6, and TNFα [92]. They further identified prostaglandin E_2_ (PGE_2_) and IL-10 abundance in ascites as a key suppressive mediator compromising the ability of DCs to prime T-cell responses in ovarian cancer. Similarly, Goyne et al. reported CD14^+^ myeloid cells, isolated from ovarian cancer ascites comprising immature DCs or monocyte-derived myeloid cells, inhibit antigen-specific CD4^+^ T-cell responses in vitro, via the secretion of IL-10 and indoleamine 2,3-dioxygenase (IDO), thereby undermining adaptive immune activation [93]. These findings suggest DCs in ascites may adopt a tolerogenic or suppressive phenotype that directly contributes to immune evasion, T-cell dysfunction, and poor anti-tumour immunity in ovarian cancer.

Multiple DC subsets participate in shaping ovarian cancer immunity. cDC1s typically characterised by CD141/BDCA3 and XCR1 are critical for the cross-presentation of tumour cell-associated antigens to CD8^+^ T cells, which then kill cancerous cells by inducing apoptosis [94]. Mounting evidence suggests that cDC1s that are required for spontaneous anti-tumour T-cell priming and responsiveness to CD137 costimulatory checkpoint blockade offer opportunities for improved cancer immunotherapy [95]. However, in ovarian cancer, these cells are significantly reduced in both tumours and ascites, correlating with the poor infiltration of cytotoxic T cells and worse prognosis [96]. A recent study found that intratumoural cDC1s are suppressed by high lactic acid concentrations, which inhibit IL-12 production and weaken CD8^+^ T-cell priming, further emphasising how metabolic features of the ovarian cancer microenvironment undermine DC function [97]. On the other hand, pDCs also play a distinct immunoregulatory role in ovarian cancer by accumulating in ascites where they adopt an immunosuppressive state. Labidi-Galy et al. reported that pDC infiltration into ovarian tumours is strongly associated with reduced overall survival, as these cells upregulate ICOS-L and drive expansion of IL-10-producing regulatory T cells [98]. They also found that tumour-associated pDCs exhibit impaired IFN-α secretion, enabling cancer cells to evade innate anti-viral and anti-tumour responses in ovarian cancer patients. These findings collectively highlight the differential and suppressive roles of distinct DC subsets within the ovarian cancer microenvironment, presenting significant challenges and potential therapeutic targets for enhancing anti-tumour immunity.

Circulating dendritic cells in ovarian cancer often exhibit impaired maturation and reduced antigen presentation capacity, reflecting systemic immunosuppression rather than immune surveillance. A study by Christoph Scholz and team found that, when monocyte-derived DCs from healthy donors are exposed to malignant ascites or to the ovarian cancer-associated glycoprotein glycodelin in vitro, the cells adopt a tolerogenic phenotype [99]. These DCs maintain antigen uptake but show diminished upregulation of costimulatory molecules, increased IL-10 production, and reduced capacity to stimulate T-cell proliferation. Similarly, both myeloid DCs (mDCs) and pDCs in peripheral blood or peritoneal fluid frequently express elevated inhibitory molecules such as PD-L1/PD-L2, leading to immunosuppression, impaired T-cell activation, and poor prognosis in ovarian cancer patients [100]. Moreover, another study found that the peritoneal fluid from both low- and high-grade ovarian cancer inhibits monocyte differentiation into functional dendritic cells, whereby the earlier induces DCs with a phenotype capable of promoting Treg differentiation while the latter promotes differentiation to an IL-10-secreting macrophage-like cell [101]. These distinct immunosuppressive phenotypes of DC underscore the need for ovarian cancer subtype-specific strategies for successful DC-based immunotherapy. These findings collectively indicate that systemic factors associated with ovarian cancer including soluble factors in blood or ascites can reprogram circulating or newly generated DCs into dysfunctional or suppressive states, thereby undermining systemic anti-tumour immunity and facilitating disease progression.

DCs in the ovarian cancer microenvironment are frequently subverted into tolerogenic phenotypes that contribute to chemoresistance by multiple, interrelated mechanisms. Tumour-conditioned DCs drive expansion and recruitment of immunosuppressive CD4^+^FOXP3^+^ Tregs and CD8^+^ suppressor T cells, thereby reducing effector anti-tumour responses and allowing chemoresistant tumour cells to stay in ovarian cancer patients [102]. Dysfunctional tumour-infiltrating DCs also promote T-cell exhaustion through deficient costimulation and upregulation of PD-L1 and ICOS-mediated signals, reducing cytotoxic T-cell responses that fail to clear chemotherapy-stressed tumour cells [103]. Moreover, tolerogenic DCs produce and sustain a suppressive cytokine milieu including TGF-β, IL-10, and VEGF that inhibits antigen presentation, supports regulatory cell networks, and fosters a microenvironment in which chemoresistant subpopulations survive and expand [104]. On the other hand, the ovarian tumour microenvironment allows DCs to be co-opted into pro-tumour roles that facilitate metastatic dissemination through angiogenesis and vascularization. Immature or tumour-associated DC precursors that are recruited to ovarian tumours can transform and differentiate into endothelial-like cells and integrate into neovasculature. A study reported that high VEGF-A/VEGFR-2 and β-defensin/CCR6 signalling drives angiogenesis that supports tumour perfusion and metastatic potential in ovarian cancer [105]. These DC-derived vascular elements help sustain blood supply, enabling tumour cell survival, invasion, and dissemination.

In conclusion, DCs are essential for antigen presentation and T-cell priming; however, in the context of ovarian cancer ascites, their function is often subverted. Instead of mounting effective anti-tumour responses, they can facilitate immunosuppression and tumour progression. Furthermore, DCs in the ovarian tumour microenvironment can paradoxically shift from sentinels of anti-tumour immunity to facilitators of chemoresistance and metastasis through immunosuppressive cell induction, T-cell anergy/exhaustion, suppressive cytokine milieu, promotion of angiogenesis, and support of tumour survival and invasion. Consequently, monitoring the prevalence and functional status of distinct DC subsets holds promise as both prognostic biomarkers and critical targets for novel immunotherapy strategies in ovarian cancer.

### 2.4. Myeloid-Derived Suppressor Cells

Myeloid-derived suppressor cells are a heterogeneous population of pathologically activated immature myeloid cells characterised by their immunosuppressive influence on innate and adaptive immune responses in the event of chronic inflammation, infection, and cancer [106,107]. The MDSCs have been primarily studied in cancer; these cells are enriched in the tumour environment and impair anti-tumour immunity. They are the major orchestrators of immune evasion, contributing to tumour progression, metastasis, and resistance to immunotherapy in the TME [108]. The MDSCs are conventionally subdivided into two broad subsets: monocytic MDSCs (M-MDSCs), resembling monocytes, and polymorphonuclear or granulocytic MDSCs (PMN-/G-MDSCs), resembling neutrophils. Human M-MDSCs are typically CD11b^+^ CD14^+^ HLA-DR^low^/^−^ CD15^−^ CD33^+^ CD126^+^, whereas G-MDSCs are CD11b^+^ CD14^−^ CD15^+^/CD66b^+^ [109]. Nevertheless, these populations differ in origin, transcriptomic signatures and dominant suppressive mechanisms, and they may interconvert or differentiate further within the TME depending on local cellular signalling [110]. Although both M-MDSCs and G-MDSCs subsets exert potent immunosuppressive effects, they employ distinct molecular mechanisms reflective of their lineage origins. G-MDSCs primarily induce antigen-specific T-cell tolerance through mechanisms such as oxidative stress and impaired antigen recognition, while M-MDSCs exert broader immunosuppressive effects by blocking T-cell responses in both antigen-specific and non-specific manners [111].

In ovarian cancer, the immune landscape is significantly shaped by the accumulation of G-MDSCs and M-MDSCs, each exhibiting distinct distribution patterns and recruitment mechanisms. G-MDSCs typically constitute the predominant population in the peripheral blood of patients, often comprising most of the total circulating MDSCs [112]. In contrast, M-MDSCs are disproportionately enriched within the TME and the ascites fluid, where they exert potent local immunosuppression [113]. This compartmentalization is driven by specific chemokine networks: the recruitment of G-MDSCs from the bone marrow is primarily orchestrated by the interaction between CXCR2 receptors and tumour-derived ligands such as CXCL, CXCL2, and CXCL8 (IL-8) [114]. Conversely, M-MDSC infiltration is largely governed by the CCL2-CCR2 and CXCL12–CXCR4 axis, where tumour-associated stromal and immune cells create chemokine gradients that guide M-MDSCs into the tumour microenvironment [115]. Both subsets share the fundamental ability to inhibit T-cell responses; however, they utilise divergent molecular machinery to achieve this suppression within the ovarian TME. G-MDSCs primarily rely on the rapid generation of reactive oxygen species (ROS) and peroxynitrite to disrupt the T-cell receptor complex, inducing antigen-specific tolerance [114]. On the other hand, M-MDSCs are characterised by the high expression of inducible nitric oxide synthase (iNOS) and ARG1; the latter depletes the microenvironment of L-arginine, a critical amino acid for T-cell proliferation, thereby arresting the cell cycle of cytotoxic lymphocytes [116]. Furthermore, M-MDSCs display a higher degree of plasticity compared to their granulocytic counterparts. Once in the ascites, M-MDSCs can rapidly differentiate into TAMs that secrete immunosuppressive cytokines like IL-10 and TGF-b, creating a self-sustaining loop of immune evasion that is more stable and long-lasting than the transient oxidative burst provided by G-MDSCs [117].

Clinically, the expansion of these MDSC subsets serves as a robust indicator of disease progression and poor therapeutic outcomes in ovarian cancer patients. Several studies have documented a consistent expansion of MDSCs in the peripheral blood, ascites, and tumour tissue of patients with EOC, compared with healthy donors or patients with benign ovarian tumours. In the comprehensive flow cytometry analysis by Okła K et al. (2019), both M-MDSC and G-MDSC subsets were significantly elevated in blood and ascites of EOC patients; notably, tumour-infiltrating M-MDSCs were associated with higher tumour grade, advanced FIGO stage, and reduced survival, suggesting that M-MDSCs may represent the most clinically relevant subset in EOC [118]. Functionally, high MDSC levels have been linked to cancer stem cell enrichment, immune evasion, and therapy resistance. In a study by Li X and co-researchers, the co-culture of patient-derived MDSCs with EOC cell lines induced sphere formation, increased colony-forming capacity, and elevated expression of stemness markers (SOX2, NANOG, OCT4a, KLF4, and c-MYC), suggesting that MDSCs directly contribute to the cancer stem cell phenotype, which is often associated with tumour recurrence and chemoresistance [119]. Separately, another study by Komura N et al. demonstrated that MDSC-derived PGE_2_ increased PD-L1 expression on ovarian cancer cells via mTOR signalling and concomitantly increased the frequency of aldehyde dehydrogenase-positive cancer stem cell-like cells; this implies that MDSCs not only suppress anti-tumour immunity, but also actively reprogram tumour cells toward more aggressive, immune-evasive phenotypes [120].

MDSCs substantially contribute to chemoresistance in ovarian cancer by reshaping both the tumour and immune microenvironment. Ascites-derived inflammatory cytokines such as IL-6 and IL-10 expand CD14^+^HLA-DR^−^/low M-MDSCs, which in turn suppress cytotoxic T-cell activity and, therefore, create a permissive niche that reduces chemotherapy-induced immunogenic cell death [116]. Mechanistically, M-MDSCs directly enhance tumour cell survival pathways by activating CSF2/p-STAT3 signalling that is promoting stemness features associated with platinum resistance in ovarian cancer [119]. Similarly, MDSC-derived PGE_2_ induces PD-L1 expression and enriches ALDH^+^ cancer stem-like cells, both of which are linked to poor responses to chemotherapy [120]. In addition, single-cell analyses further indicate that ascites create a myeloid-dominant ecosystem that maintains immunosuppressive and pro-survival programmes in metastatic tumour cells, providing an optimal environment for chemoresistance [48]. Clinically, higher circulating and tumour-infiltrating MDSC levels correlate with advanced disease and poorer outcomes, consistent with their role in promoting resistance to frontline therapies [118]. Together, these findings indicate that MDSCs not only suppress anti-tumour immunity but also directly reprogram ovarian cancer cells toward phenotypes that resist chemotherapy-induced cell death.

Beyond immunosuppression and chemoresistance, MDSCs contribute to metastatic progression in ovarian cancer. MDSCs are recruited to the TME and pre-metastatic niches by chemoattractant signals. In ovarian cancer, a tumour-derived complement component C5 (C5), which is upregulated via a metastasis-associated long non-coding RNA (LncOVM)–PPIP5K2 axis, has been shown to attract MDSCs into the TME, and disruption of this C5–MDSC recruitment reduces metastasis in xenograft model [121]. Once localised, MDSCs may facilitate formation of a permissive metastatic niche by remodelling extracellular matrix, promoting angiogenesis, and suppressing anti-tumour immunity, although the detailed molecular mediators in ovarian cancer remain incompletely defined [122]. Furthermore, MDSC accumulation in malignant ascites may support the survival and dissemination of multicellular tumour spheroids, which are believed to contribute to peritoneal spread and distant metastasis [113].

In conclusion, current evidence supports that M-MDSCs and G-MDSCs are central regulators of ovarian cancer progression through multifaceted roles: promoting immunosuppression, enhancing cancer cell stemness and survival, driving chemoresistance, and facilitating metastasis. Their consistent correlation with poor clinical outcomes and resistance to standard therapies underlines their value as both prognostic biomarkers and therapeutic targets.

## 3. Therapeutic Strategies Targeting Monocytes and Their Derivatives in Ovarian Cancer

Monocytes and their derivatives play central roles in ovarian cancer progression, shaping immunosuppression, chemoresistance, and metastatic potential. Therapeutic strategies targeting these myeloid populations are therefore emerging as a promising opportunity to improve patient outcomes. Monocytes serve as precursors to TAMs and immunosuppressive MDSCs, whereby their recruitment and differentiation are driven by key pathways such as CSF-1/CSF-1R and CCL2/CCR2. Macrophages exhibit remarkable plasticity, with pro-tumourigenic M2-like TAMs promoting immunosuppression, angiogenesis, and chemotherapy resistance, while M1-like TAMs enhance anti-tumour immunity. Recent advances have explored reprogramming M2-like TAMs toward M1 phenotypes using immunomodulatory agents, CD47/CD40 dual blockade, CAR-macrophages, or TREM-2-targeting antibodies. Similarly, MDSC-targeted approaches including depletion, functional inhibition, recruitment blockade, and promotion of maturation have shown efficacy in preclinical and early clinical studies. In addition, DC-based vaccines, such as DCVAC/OvCa, demonstrate that effective T-cell priming requires simultaneous modulation of suppressive myeloid cells to overcome the hostile tumour microenvironment. To provide an integrated view of how monocytes and their derivatives can be therapeutically manipulated in ovarian cancer, Table 2 summarises the major strategies currently under investigation. These approaches span monocyte recruitment blockade, macrophage repolarization, dendritic cell activation, and MDSC depletion or reprogramming, highlighting both preclinical advances and emerging clinical applications. The table consolidates the mechanistic basis and therapeutic rationale for each strategy, offering a concise reference for understanding how myeloid-directed interventions are reshaping the treatment landscape. Collectively, these approaches underscore a multifaceted strategy by integrating myeloid targeting, immunomodulation, and combination therapies to overcome immune evasion and enhance therapeutic responses in ovarian cancer.

### 3.1. Targeting Monocyte Recruitment

Targeting monocyte recruitment, differentiation, and functional polarisation represents one of the most promising strategies to improve ovarian cancer treatment outcomes. A widely studied approach involves inhibiting the CSF-1R axis. The CSF-1 and its receptor, CSF-1R, form a crucial signalling pathway that drives the recruitment, survival, and differentiation of monocytes and macrophages, particularly within the ovarian TME. Tumour cells secrete high levels of CSF-1 to draw circulating monocytes into the tumour site. Once in the tumour, the binding of CSF-1 to CSF-1R activates intracellular downstream cascades that not only promote monocyte survival but also drive their differentiation into pro-tumourigenic M2-like TAMs. This process establishes a self-prolonging, immunosuppressive cycle that facilitates tumour growth and metastasis [9]. Since the CSF-1/CSF-1R pathway is crucial for the survival of ovarian cancer, inhibiting the CSF-1R could be the key therapeutic approach. This can be achieved using small-molecule inhibitors like pexidartinib (PLX3397) or anti-CSF-1R monoclonal antibodies like emactuzumab and AMG 820 [9,16,123]. These agents reduce the infiltration of new monocytes into the tumour and can lead to the death of existing TAMs. In a recent preclinical study, the combinatorial treatment of PLX3397 with paclitaxel significantly suppressed ovarian cancer cell proliferation in vitro, reduced tumour burden in vivo, and reprogrammed the tumour microenvironment by reducing immunosuppressive macrophage populations [16]. A phase 1 clinical trial study of AMG 820 (NCT01444404) in patients with advanced solid tumours, which included ovarian cancer patients, demonstrated that the drug was well tolerated, and successfully reduced macrophages in skin biopsies paved the way to the initiation of phase Ib/II trial (NCT02713529) investigating the combination of AMG 820 and the PD-1 inhibitor pembrolizumab in patients with advanced solid tumours [123].

The chemokine CCL2 and its receptor CCR2 on circulating monocytes also mediate monocyte trafficking into tumour tissue [124]. A recent in vivo study on combination of a CCR2 inhibitor (BMS CCR2 22) and bevacizumab demonstrated sustained anti-cancer effects in patient-derived ovarian cancer models, achieving significant growth suppression in both bevacizumab-resistant and -sensitive tumours [125]. This enhanced efficacy was linked to the combination’s ability to suppress angiogenesis effectively than bevacizumab alone, targeting microvessels by inhibiting the CCR2B-MAPK pathway. Moreover, carlumab, an human anti-CCL2 IgG1κ monoclonal antibody, was investigated in a phase 1 study (NCT00537368) involving patients with advanced solid malignancies reported that one patient with ovarian cancer showed a >50% reduction in CA125 and achieved stable disease for 10.5 months [126]. However, carlumab as a single agent later faced challenges due to difficulty in achieving sustained suppression of free CCL2, leading to limited overall efficacy in later-stage trials [127]. Therefore, the combined blockade of both recruitment (CCL2/CCR2) and survival/differentiation (CSF-1/CSF-1R) may produce synergistic effects, and minimising the replenishment of TAMs from circulating monocytes has high potential to enhance anti-tumour immune response in treating ovarian cancer.

### 3.2. Macrophage Reprogramming

Another strategy in the management of ovarian cancer focuses on reprogramming pro-tumourigenic M2-like macrophages into an anti-tumourigenic M1-like state. While M2-like macrophages promote immunosuppression and angiogenesis, M1-like macrophages are essential for an effective anti-cancer immune response. A past research work has highlighted the remarkable plasticity of TAMs and the potential for immunomodulatory drugs and epigenetic regulators to induce this phenotypic switch. This process involves two primary mechanisms: immunomodulatory signalling and metabolic reprogramming. M2-like macrophages can be reprogrammed using pro-inflammatory signals, such as IFN−γ or TLR agonists, which trigger the polarisation shift. These signals then activate specific intracellular pathways, such as STAT1, which upregulate M1-associated genes responsible for producing pro-inflammatory cytokines like IL-12 and TNF-α [70]. Moreover, several in vitro and in vivo studies provide strong support for the efficacy of this reprogramming approach. For example, a 2019 study showed that, by using targeted nanocarriers to deliver specific mRNAs to macrophages in mice with ovarian cancer, researchers could successfully induce a phenotypic shift from M2 to M1. This reprogramming led to a significant decrease in immunosuppressive macrophages (from an average of 43% in controls to 2.5% in treated mice) and a corresponding increase in M1-like macrophages (from 0.5% to 10.2%). This led to a dramatic reduction in tumour growth, with in vivo disease regression and long-term survival achieved in 40% of the treated animals [17]. Similarly, another study using patient-derived ovarian cancer organoids showed that M2 macrophages, but not M1 macrophages, increased the viability of the cancer cells and made them less sensitive to paclitaxel chemotherapy. However, when the cells were treated with BMS-777607 to repolarise the M2 macrophages in vitro, it successfully reduced the viability of the cancer organoids in a macrophage-dependent manner. This finding was further validated in a humanised patient-derived xenograft mouse model where repolarisation of M2 macrophages, combined with paclitaxel, reduced tumour burden with no sign of regrowth [128].

In a recent clinical study, SL-172154 (NCT04406623) demonstrated that dual CD47 blockade and CD40 agonism are both biologically active and clinically feasible in patients with platinum-resistant ovarian cancer [129]. In this first-in-human study, treatment produced clear pharmacodynamic evidence of immune activation, including near-complete target engagement at higher doses, cyclical induction of inflammatory cytokines, and a notable shift in the tumour microenvironment toward an M1-dominant macrophage phenotype accompanied by increased CD8^+^ T-cell infiltration. These findings indicate successful reprogramming of immunosuppressive tumour-associated macrophages and enhanced recruitment of effector lymphocytes showing great potential to tackle two central obstacles in ovarian cancer immunotherapy. Clinically, SL-172154’s immune-modulating effects and manageable safety profile provide a promising foundation for continued development, especially in combination regimens aimed at translating this myeloid-targeted activation into more durable anti-tumour responses. On the other hand, chimeric antigen receptor macrophage (CAR-M) therapy is emerging as a promising cellular strategy for ovarian cancer, and recent clinical evidence underscores both its safety and its potential to reshape the tumour microenvironment. In the first-in-human study (NCT06562647) of autologous mesothelin-targeted CAR-M in recurrent ovarian cancer, patients tolerated the therapy well, with no dose-limiting toxicities and only low-grade, manageable adverse events, demonstrating that macrophage engineering is clinically feasible [130]. The infused CAR-Ms persisted transiently, trafficked to the tumour site, and showed evidence of switching the local immune landscape toward a more pro-inflammatory, antigen-presenting state highlighting a mechanism distinct from CAR-T cells, which rely more on direct cytotoxicity. Although objective tumour regressions were limited, this study provides a crucial proof of concept that reprogrammed macrophages can overcome some of the profound immunosuppressive barrier’s characteristic of ovarian cancer. Looking ahead, future development will likely focus on enhancing CAR-M persistence, optimising antigen targets beyond mesothelin, combining CAR-M with checkpoint inhibitors or myeloid-modulating agents, and leveraging allogeneic or off-the-shelf platforms to improve accessibility. Together, these advances position CAR-Ms as a novel and adaptable arm of next-generation cell therapy for ovarian cancer. These results collectively highlight that phenotypic changes in macrophages can directly impair tumour growth and overcome chemotherapy resistance, offering a powerful new avenue for ovarian cancer treatment.

Besides this, TREM-2-targeting strategies are gaining momentum to reprogram immunosuppressive macrophages in ovarian cancer, and recent evidence from the PY159 and PY314 development programme highlights their therapeutic promise. In the study, (NCT04682431, NCT04691375) both PY159 and PY314 engineered antibodies designed to modulate TREM-2-expressing myeloid cells demonstrated the ability to shift macrophages away from a dysfunctional, immunosuppressive phenotype commonly enriched in platinum-resistant ovarian tumours [131]. By engaging TREM-2, these agents enhanced antigen-presenting functions, increased pro-inflammatory signalling, and promoted a microenvironment more conducive to T-cell activation. Early clinical evaluation showed encouraging signals of biological activity, including remodelling of the myeloid compartment and evidence of immune re-engagement, while maintaining a manageable safety profile. Although objective responses were limited in this heavily pre-treated population, the trial provides important proof that TREM-2 is a viable therapeutic node for targeting tumour-associated macrophages. Collectively, these findings suggest that TREM-2-directed therapies have high potential when integrated into combination regimens with checkpoint blockade agents that further enhance myeloid plasticity, thus, representing a promising direction for future ovarian cancer immunotherapy.

Reprogramming the phenotype of immunosuppressive and pro-tumourigenic M2-like TAMs into an anti-tumourigenic M1-like state represents a crucial advancement in ovarian cancer immunotherapy. This approach is highly effective because M1-like macrophages enhance anti-tumour immunity by producing pro-inflammatory cytokines and promoting T-cell recruitment and activation in ovarian malignancy. Furthermore, by reversing the immunosuppressive TME, TAM reprogramming agents such as CSF-1R inhibitors, TREM-2 modulators, and novel CAR-Ms effectively synergize with immunotherapies and even enhance the efficacy of chemotherapy, offering a potent combination approach to overcome the profound resistance barriers characteristic of advanced ovarian cancer.

### 3.3. Targeting Myeloid-Derived Suppressor Cells (MDSCs)

Myeloid-derived suppressor cells are attractive therapeutic targets in ovarian cancer due to their prominent driving role in immunosuppression, stemness, and chemoresistance within the TME. Targeting MDSCs in ovarian cancer has progressed along four main strategies which are (1) depleting MDSCs, (2) inhibiting their immunosuppressive functions, (3) blocking their recruitment to the TME, and (4) promoting their maturation into non-suppressive myeloid populations, each supported by preclinical and translational evidence. Depletion studies in ovarian cancer mouse models using anti-Gr-1 antibodies reduced ascites, tumour burden, and improved survival [132,133]. Antibodies against GM-CSF or blockade of GM-CSF signalling likewise diminished MDSC expansion and tumour progression in another preclinical work [133]. Furthermore, blocking recruitment through CXCR2 or CXCR4 antagonists impaired MDSC trafficking in mouse models [115] and blocking PGE_2_/COX-2 signalling reduced CXCR4 expression and curtailed MDSC accumulation within the ovarian tumour milieu [114]. Moreover, a study reports repurposing the antidiabetic drug metformin, and it decreased CD39/CD73 expression and abrogated MDSC suppressive function in ovarian cancer patient samples and preclinical studies [134]. A pre-clinical study showed that the RPN13/ADRM1 inhibitor RA190 reversed MDSC-mediated suppression by reducing the expression of STAT3, arginase, iNOS, and IL-10 and boosting IL-12 in MDSCs, improving CD8^+^ T-cell responses and tumour control in mice bearing syngeneic ovarian tumour [135]. Moreover, another study on ID8 ovarian cancer mouse model demonstrated inhibition of thrombin by dabigatran etexilate, enhanced cisplatin efficacy, decreased the number of Gr1+/CD11b+ MDSCs, and alleviated MDSC-associated immunosuppression by reducing TGF-β, VEGF, IL-6, IL-10, and MCP-1 in the ascites [136]. On the other hand, targeting IL-10 signalling reprogrammed MDSCs, increased T-cell activity, and prolonged survival in ovarian cancer models [137]. The therapeutic targeting MDSCs is essential for reducing the immunosuppressive environment of ovarian cancer. Pursuing strategies like depletion, functional inhibition, and recruitment blockade, MDSC-targeting agents directly reduce immunosuppression by lowering suppressive factors and successfully restoring CD8^+^ T-cell activity. Crucially, this alleviation of immune suppression acts as a powerful synergist for other immunotherapies, enhancing the efficacy of both TAM reprogramming by clearing suppressive hurdles that affect macrophage polarisation and DC vaccination by preventing the MDSC-mediated deactivation of the CD8^+^ T cells primed by the vaccine. Together these approaches offer rational combination pathways with immunotherapy and chemotherapy, warranting prioritised clinical translation targeting MDSCs in ovarian cancer.

### 3.4. Dendritic Cell (DC) Therapies

Dendritic cells are being actively targeted in ovarian cancer to restore antigen presentation and revitalise anti-tumour T-cell responses. DC vaccine strategies that have been studied earlier using autologous, tumour-lysate-pulsed DCs demonstrated safety and induced measurable tumour-specific CD8^+^/CD4^+^ T-cell responses in recurrent ovarian cancer patients [138]. A recent DCVAC/OvCa vaccine, with autologous dendritic cells pulsed with killed ovarian cancer cells and matured by the TLR3 ligand ex vivo, demonstrated a favourable safety profile, with few serious adverse events that were not associated with significant safety concerns attributable to DC vaccination or leukapheresis in patients undergoing first-line chemotherapy (carboplatin + paclitaxel) after debulking surgery [139]. In this study (NCT02107937), DCVAC/OvCa was evaluated either concurrently with chemotherapy, sequentially after chemotherapy, or against chemotherapy alone. Progression-free survival was the longest in the sequential-vaccination group (not reached), which achieved a statistically significant improvement with a hazard ratio (HR) of 0.39 (95% CI = 0.16–0.96; *p* = 0.0336) compared with chemotherapy alone (21.4 months). In contrast, adding the vaccine concurrently with chemotherapy (20.3 months) did not affect progression-free outcomes. Moreover, the HR for concurrently with chemotherapy versus chemotherapy alone group was 0.98 (95% CI = 0.48–2.00; *p* = 0.9483), further indicating no benefit from concurrent administration. Median OS was not reached in any group after a median follow-up of 66 months, with 34% of events recorded, although modest trends favoured both vaccine groups. However, in the platinum-sensitive, relapsed setting (SOV02) (NCT02107950), the addition of DCVAC/OvCa to carboplatin and gemcitabine did not significantly improve PFS (HR = 0.73, *p* = 0.274), but OS was significantly prolonged by approximately 13.4 months (HR = 0.38, *p* = 0.003) compared with chemotherapy alone [140]. Collectively, these findings indicate that administering DCVAC/OvCa after completion of platinum-based first-line therapy provides the greatest clinical benefit, particularly through a meaningful extension of progression-free survival. Interestingly, plasmacytoid DCs in ovarian tumours adopt tolerogenic phenotypes, upregulate ICOS-L, and foster regulatory T-cell expansion, further impeding anti-tumour immunity [104]. Despite promising immunologic readouts, objective response rates remain modest, reflecting barriers of immune suppression and poor T-cell homing. Moving forward, rational combinations that neutralise suppressive cytokines, inhibit myeloid checkpoints, and enhance DC maturation should be prioritised in trials. Integrating biomarker-driven patient selection will accelerate translation of DC-based therapies into meaningful clinical benefit for ovarian cancer patients.

Consequently, to translate the proven immunological activity of DC vaccines into consistent clinical efficacy in ovarian cancer, these therapies are fundamentally dependent on effective myeloid modulation. This synergy arises because highly prevalent immunosuppressive cells TAMs and MDSCs can suppress DC function and diminish the effect of primed T cells, thereby creating a hostile microenvironment. Therefore, targeting and reducing the numbers and suppressive functions of TAMs and MDSCs synergizes with DC-based immunotherapy; by clearing the suppressive hurdles, myeloid modulation enhances the quantity and quality of T cells generated by DC vaccines, maximising the therapeutic window and offering the greatest potential for durable anti-tumour responses. Therefore, multifaceted strategies targeting monocytes and their derivatives highlight a paradigm shift towards a more comprehensive approach in the management of ovarian cancer.

## 4. Prognostic Significance of Monocytes in Ovarian Cancer

Monocytes are emerging as valuable diagnostic and prognostic tools beyond their therapeutic significance for ovarian cancer. Given that the number and phenotype of circulating monocytes change in response to a developing tumour, they provide a non-invasive way to detect the disease and monitor its progression. Traditional markers like CA-125 and HE4 have well-known limitations, including poor sensitivity in early-stage disease and a lack of specificity, as their levels can also be elevated in benign conditions [141,142]. In contrast, several studies have identified specific subsets of circulating monocytes, particularly intermediate monocytes and a subpopulation of monocytes defined by their expression of HLA-DR, that show a robust expansion in patients with ovarian cancer [43,143]. A study by Prat et al. demonstrated a significant increase in the percentage of intermediate monocytes in patients with ovarian cancer compared to healthy controls, with the percentage rising from an average of 3.5% in controls to over 6.0% in patients with advanced-stage disease [43]. This expansion has been shown to correlate with both tumour burden and disease stage, suggesting that these specific monocyte populations could serve as a more reliable indicator than traditional markers alone [144]. Ovarian cancer is often diagnosed at an advanced stage, leading to high recurrence rates and a low five-year survival rate. EOC accounts for over 90% of cases, emphasising the need for effective prognostic markers [145]. Classic indicators like residual tumour size and platinum sensitivity are primarily available postoperatively, but focus has shifted to more accessible markers reflecting the systemic biological state [18,19,146]. Recent oncological research has increasingly recognised systemic inflammation as a core “hallmark of cancer,” playing a pivotal role in tumour initiation, growth, and metastasis [147]. This understanding has prompted investigations into easily accessible, non-invasive, and cost-effective inflammatory biomarkers derived from routine complete blood counts [23].

A meta-analysis of over 2200 patients found that a low LMR was significantly associated with both poor OS and PFS [148]. In the same study, the pooled HR for OS was 1.92 (95% CI: 1.58–2.34, *p* < 0.001), while the HR for PFS was 1.70 (95% CI: 1.54–1.88, *p* < 0.001), indicating that patients with a low LMR had a nearly 2-fold increased risk of mortality and disease progression compared to those with a high LMR. A different study also found that a cutoff LMR value of 4.65 was the most effective [145]. This study showed that patients with an LMR below this value had a significantly shorter median OS (48.5 months) compared to those with an LMR above this value (105.9 months). Thus, LMR can be a useful tool for predicting patient outcomes. The LMR and MLR are reciprocal ratios that quantify the balance between two key immune cell populations: lymphocytes, which primarily mediate anti-tumour immunity, and monocytes, which can be reprogrammed by the tumour to promote its progression [144]. A high LMR, which is equivalent to a low MLR, is generally considered a favourable prognostic indicator, suggesting a robust host anti-tumour immune response relative to pro-tumour inflammatory activity [20]. The LMR is calculated by dividing the absolute lymphocyte count by the absolute monocyte count, while the MLR is calculated by dividing the absolute monocyte count by the absolute lymphocyte count, which can be derived from a standard blood profile.

The ease of measurement makes these markers particularly attractive for clinical application, especially in resource-limited settings. Nevertheless, the lack of a standardised, universally accepted cutoff value of LMR and MLR remains a significant challenge in clinical application. Researchers typically use statistical methods, such as receiver operating characteristic (ROC) curves, to determine an optimal threshold for their specific patient cohort, leading to a wide variation in reported values across different studies. For instance, a 2023 study on EOC identified an optimal LMR cutoff of 4.65, while a meta-analysis of other studies on ovarian cancer reported a range of cutoff values from 1.85 to 4.35 [20,145]. Similarly, MLR cutoff values have been reported to range between 0.23 and 0.54, with a median of 0.26, and one study identified an optimal MLR cutoff of 0.38 [20,21]. The variability in reported MLR/LMR cutoff values across studies may also arise from differences in measurement techniques and instrumentation used. Automated haematology analysers from different manufacturers (e.g., Sysmex, Beckman Coulter, Abbott) differ in detection principles, calibration algorithms, and gating strategies, leading to inter-platform variability in absolute leukocyte counts [149,150]. In addition, pre-analytical factors such as blood withdrawal timing, anticoagulant choice, sample storage, or delays before processing can also influence measured leukocyte values [151]. Moreover, heterogeneity in patient populations, including disease stage, inflammation burden, or comorbidities, yields differing baseline immune profiles, which may also shift the distributions of LMR/MLR in the study cohorts. Finally, methodological differences in cutoff derivation, like using ROC curves with Youden index, equal sensitivity–specificity point, minimum distance criterion, or median splits, could also contribute to the additional discrepancies in thresholds [152]. This variability prevents the establishment of a single and definitive threshold for clinical use. Despite this heterogeneity, the overall prognostic trend is consistent, whereby a low LMR or a high MLR is consistently associated with worse clinical outcomes in ovarian cancer. Therefore, a deeper look into the biological rationale of LMR or MLR in ovarian TME warrants further discussion.

The prognostic power of the LMR/MLR is orchestrated in the opposing functions of its two cellular components within the TME. Lymphocytes, including tumour-infiltrating lymphocytes, are central to the host’s immune defence against cancer. Meanwhile, activated cytotoxic T cells, T helper cells, and B cells work in concert to secrete inflammatory cytokines that induce direct cytotoxic cell death, and eventually inhibit tumour cell proliferation [153]. A high absolute lymphocyte count is thus indicative of a vigorous and effective anti-tumour immune response [154]. In contrast, circulating monocytes are recruited to the TME and differentiate into TAMs. These macrophages, rather than fighting the tumour, can be “re-educated” by tumour cells to adopt a pro-tumourigenic phenotype [144]. TAMs promote tumour growth by stimulating angiogenesis, facilitating invasion and metastasis, and actively suppressing the anti-tumour activity of T cells, thereby fostering an immune-tolerant state [22]. Consequently, a high monocyte count can be an indirect but powerful marker of a tumour’s ability to manipulate its environment and progress. The LMR/MLR provides a more comprehensive view of the systemic immune landscape than either cell counts alone by integrating the host’s anti-tumour response (lymphocytes) with the pro-tumour inflammatory state (monocytes). A low LMR or high MLR suggests an imbalance where the pro-tumour influence of monocytes outweighs the anti-tumour efforts of lymphocytes, a condition that favours cancer progression and resistance to therapy [148]. This biological mechanism is validated by a deeper examination of the individual cell counts among ovarian cancer patients. A 2025 study on ovarian tumours has shown that the negative prognostic value of low LMR appears to be driven predominantly by the monocyte component [155]. The study found a significant increase in the absolute monocyte count in malignant cases, while the absolute lymphocyte count showed no statistically significant difference. This suggests that the predictive power of LMR/MLR in ovarian cancer is primarily a consequence of the increased influx or expansion of pro-tumourigenic monocytes, rather than an overall depletion of anti-tumour lymphocytes. This association provides a crucial biological explanation for the ratio’s prognostic significance in ovarian malignancy.

Recent studies and meta-analyses consistently reinforce the finding that a low LMR is an independent predictor of poor OS and PFS in patients with EOC, as summarised in Table 3. The predictive value of LMR/MLR is not unique to ovarian cancer, as a low LMR or high MLR is consistently associated with a poor prognosis across a wide range of malignancies, including pancreatic, lung, and colorectal cancers [24,156,157]. This cross-cancer consistency underscores that the fundamental biological relationship between systemic immune balance and cancer outcomes is a broadly applicable principle. A retrospective cohort study involving 368 EOC patients demonstrated that a low LMR (cutoff value of 4.65) was an independent factor for predicting poor OS, with an HR of 1.49 (*p* = 0.041) [145]. Another meta-analysis of 12 studies, including over 3300 ovarian cancer cases, confirmed that a high LMR was associated with a favourable prognosis, showing a pooled HR of 1.85 (95% CI: 1.50–2.28, *p* < 0.001) for OS and an HR of 1.70 (95% CI: 1.49–1.94, *p* < 0.001) for PFS [20]. In addition, a separate study from 2018 by Marc Cucurull and colleagues specifically examined the prognostic value of MLR in EOC [21]. They found MLR was a laboratory parameter that significantly impacted OS (*p* = 0.02) and was an independent prognostic factor and identified an optimal MLR cutoff of 0.38. Patients in the high-MLR group (MLR > 0.38) had a significantly higher risk of death (HR = 2.9, 95% CI: 1.3–6.5) compared to those in the low-MLR group (MLR ≤ 0.38). The median OS for the high-MLR group was 35 months, which was substantially shorter than the 100 months for the low-MLR group. Furthermore, a recent umbrella review of systematic reviews and meta-analyses also demonstrated that a high baseline MLR was an independent predictor of poor OS and PFS in patients with EOC [26].

A low LMR is not only an independent prognostic factor for survival but is also significantly correlated with other established indicators of aggressive disease. Studies have shown a strong association between low LMR and advanced FIGO stage as well as poor tumour differentiation as observed in histopathological results [26,145]. Moreover, a significant negative correlation (r = −0.28, *p* = 0.02) has also been found between LMR and serum CA-125 levels, indicating that lower LMR values are associated with a higher tumour burden and more advanced disease [144]. On top of that, a low LMR is also associated with the presence of ascites and metastasis, further underscoring its utility as a marker of aggressive tumour biology [26]. The prognostic utility of LMR/MLR is not static but can be influenced by other clinical factors and treatment interventions. Subgroup analyses have revealed that a low LMR is particularly predictive of a poor prognosis in specific patient populations, including those who have undergone optimal surgery and those who complete adjuvant chemotherapy [145]. Patients with a low LMR who completed adjuvant chemotherapy had a substantially longer median OS (105.9 months) compared to those who did not (48.5 months). This suggests that the LMR/MLR can be used as a valuable tool for risk stratification, helping to identify patients who may derive the greatest benefit from aggressive or complete treatment regimens.

In summary, the LMR/MLR offers a potential clinically valuable, non-invasive, and cost-effective prognostic biomarker in ovarian cancer. A low LMR or high MLR is consistently associated with an aggressive tumour phenotype, advanced disease stage, and poor overall and progression-free survival. Predictive power is highly dependent on the imbalance between pro-tumour monocytes and anti-tumour lymphocytes, an effect that appears to be primarily driven by the monocyte component. Despite the compelling evidence, a critical analysis of the existing literature reveals significant limitations that must be addressed for LMR/MLR to be adopted as a standard clinical tool. A meta-review of the topic explicitly criticised the quality and redundancy of existing meta-analyses, noting that many were based on retrospective cohorts with small sample sizes. The lack of a standardised cutoff value further complicates its clinical application and underscores the need for more rigorous research. Future studies should prioritise large-scale, prospective clinical trials with standardised methodologies and multicentre collaboration to establish reliable and generalizable findings. Ultimately, the integration of LMR/MLR with other clinical and molecular markers in a dynamic, predictive model holds the greatest promise for improving risk stratification and personalising treatment strategies for patients with ovarian cancer.

## 5. Conclusions and Future Perspectives

The integration of monocyte-based biomarkers and therapeutic strategies into clinical practice will require well-designed prospective studies and standardised methodologies. Establishing universally accepted cutoff values for LMR and MLR and validating monocyte subsets as diagnostic and prognostic indicators are essential steps toward clinical translation. In parallel, combining monocytes, TAMs, MDSCs, and DCs-targeted therapies with chemotherapy or immunotherapy holds strong potential to overcome resistance and improve patient outcomes. Future research should prioritise multicentred collaborations, larger patient cohorts, and the incorporation of single-cell technologies to capture immune heterogeneity more accurately. Finally, translating these advances into clinical tools may enable earlier diagnosis, more precise risk stratification, and the development of tailored treatment strategies for ovarian cancer patients.

## Figures and Tables

**Table 1:** Overview of recruitment, polarisation, immunosuppressive activity, chemoresistance mechanisms, and metastatic roles of monocyte-derived cell subtypes in ovarian cancer.

Cell Type	Physiological Functions/ Introduction	Recruitment/ Differentiation in OC	Immunosuppressive Roles	Contribution to Chemoresistance	Contribution to Metastasis	Refs.
Monocytes	- Major circulating myeloid cells (~10% of leukocytes) - Precursors for macrophages, DCs, and M-MDSCs - Classified into classical, intermediate, and non-classical subsets with distinct functions	- Recruited by CCL2 and CXCL12 into tumours/omentum - Main precursors for TAM differentiation	- Produce IL-10 and TGF-β2 in ascites - Suppress T-cell proliferation of intermediate monocytes associated with low effector/regulatory T-cell ratio - Tumour-derived EVs and factors induce M2-like programming	- TAMs derived from monocytes promote platinum resistance via CCL2-mediated CSC maintenance - TAMs enhance DNA repair via Pol-η; CD163^+^ TAM exosomes (miR-221-3p) promote EMT and drug resistance - Monocyte–TAM crosstalk supports survival of chemotherapy-stressed cells	- Support pre-metastatic niche formation (CD163^+^Tim4^+^ macrophages) - Promote ECM remodelling - Exosomal miR-221-3p drives EMT - Omental macrophages recruit monocytes to metastatic sites	[9,30,31,32,33,34,35,36,37,38,39,40,41,42,43,44,45,46,47,48,49,50,51,52]
Macrophages	- Innate immune cells involved in defence regulation and tissue remodelling - Polarise into M1 and M2 subtypes - M2 subtypes include M2a–M2d with specialised anti-inflammatory/remodelling functions	- Recruited via CCL2 and CSF-1 from peripheral monocytes - Differentiate into TAMs under TME signals - M2 polarisation driven by IL-4, IL-10, TGF-β via JAK/STAT6, PI3K/AKT/mTOR, and TGF-β/SMAD pathways	- M2-like TAMs secrete IL-10 and TGF-β, suppressing cytotoxic T cells and NK cells - Attract and activate Tregs, intensifying immunosuppressive environment - High IL-10/TGF-β correlates with poor OS in OC	- TAMs activate NF-κB and STAT3 in cancer cells, promoting survival - High M2 density linked to platinum resistance, shorter PFS, and poor OS - M1/M2 ratio affects treatment efficiency	- M2-TAMs secrete MMPs degrading ECM, creating invasion pathways - Secrete CCL6/CCL23 and CCL9/CCL5 to attract OC cells and facilitate omental metastasis - Serve as co-travellers and support metastatic colonisation	[25,53,54,55,56,57,58,59,60,61,62,63,64,65,66,67,68,69,70,71,72,73,74,75,76,77,78,79,80,81,82,83,84]
DCs	- Professional APCs - Initiate innate and adaptive immunity - cDC1, cDC2, and pDC subsets with specialised T-cell priming functions	- Malignant ascites inhibit DC maturation - Ascites and tumour factors reprogram circulating and monocyte-derived DCs into tolerogenic phenotypes - cDC1 and pDC levels/function suppressed in OC	- Ascite-derived factors (PGE_2_, IL-10) impair DC activation and IL-12/IL-6/TNFα production - DCs induce Tregs via ICOS-L, IL-10, and IDO - Express PD-L1/PD-L2; exhibit impaired IFN-α secretion - Promote T-cell exhaustion	- Tolerogenic DCs expand Tregs and CD8^+^ suppressor cells - PD-L1/ICOS signalling promotes T-cell dysfunction - Suppressive cytokines (IL-10, TGF-β, VEGF) foster survival of chemoresistant tumour cells	- Tumour-associated DC precursors differentiate into endothelial-like cells supporting angiogenesis - VEGF-A/VEGFR-2 and β-defensin/CCR6 signalling promote vascularization and metastatic spread	[85,86,87,88,89,90,91,92,93,94,95,96,97,98,99,100,101,102,103,104,105]
MDSCs	- Immature, immunosuppressive myeloid cells - Key drivers of immune evasion - Divided into M-MDSCs and G-MDSCs with distinct phenotypes and mechanisms	- G-MDSCs recruited via CXCR2 ligands (CXCL1/2/8) - M-MDSCs enriched in tumour/ascites and recruited by CCL2-CCR2 and CXCL12-CXCR4 - M-MDSCs can differentiate into TAMs	- G-MDSCs suppress T cells via ROS/peroxynitrite - M-MDSCs suppress via iNOS, Arg-1, IL-10, and TGF-β - M-MDSCs form stable immunosuppressive niches - Expansion in blood and ascites correlates with tumour burden and T-cell suppression	- IL-6/IL-10 expand M-MDSCs - M-MDSCs activate CSF2/p-STAT3 to enhance stemness and platinum resistance - MDSC-derived PGE_2_ upregulates PD-L1 and enriches ALDH^+^ CSC-like cells - Ascites ecosystem maintains pro-survival programmes	- C5–MDSC recruitment axis promotes metastasis - MDSCs remodel ECM and support angiogenesis - MDSC accumulation sustains tumour spheroid survival in ascites	[48,106,107,108,109,110,111,112,113,114,115,116,117,118,119,120,121,122]

**Table 2:** Overview of therapeutic approaches targeting monocytes and their myeloid derivatives in ovarian cancer.

Cell Type	Therapeutic Strategy	Key Findings	Evidence Type	Refs.
Monocyte	Blocking Monocyte Recruitment: CSF-1/CSF-1R axis	- CSF-1/CSF-1R regulates monocyte recruitment, survival, and differentiation into M2 TAMs. - Inhibition reduces monocyte influx and depletes TAMs. - PLX3397 + paclitaxel reduced tumour burden and suppressed immunosuppressive macrophages in preclinical models. - AMG 820 trial reduced macrophages and supported combination testing with pembrolizumab.	Preclinical + Clinical (NCT01444404) (NCT02713529)	[9,16,123]
	Blocking Monocyte Recruitment: CCL2/CCR2 axis	- CCR2 inhibitor + bevacizumab suppressed tumour growth in PDX models, including bevacizumab-resistant tumours. - Carlumab showed CA-125 reduction and stable disease but lacked sustained CCL2 suppression in later trials.	Preclinical + Clinical (NCT00537368)	[124,125,126,127]
Macrophage	Macrophage Reprogramming: M2 → M1 phenotype	- IFN-γ, TLR agonists, and STAT1 activation drive M1 reprogramming. - Nanocarrier–mRNA delivery shifted M2→M1, reduced tumour growth, and prolonged survival. - BMS-777607 reversed M2-mediated chemoresistance in organoids and mouse models.	Preclinical	[17,70,128]
	Dual CD47 blockade + CD40 agonism (SL-172154)	- Induced inflammatory cytokines, increased M1-dominant phenotype, and enhanced CD8^+^ T-cell infiltration. - Demonstrated clinical feasibility and immune activation in platinum-resistant OC.	Clinical (NCT04406623)	[129]
	CAR-Macrophage (CAR-M) Therapy	- CAR-Ms trafficked to tumour, remodelled TME to pro-inflammatory state, and showed proof-of-concept activity with manageable adverse effects.	Clinical (NCT06562647)	[130]
	TREM-2 Targeting (PY159, PY314)	- Reprogrammed dysfunctional macrophages, enhanced antigen presentation, increased T-cell activation, and remodelled myeloid compartment with acceptable safety.	Early Clinical (NCT04682431) (NCT04691375)	[131]
MDSCs	Targeting MDSCs: Depletion	- Anti-Gr-1 and anti-GM-CSF antibodies reduced ascites, tumour burden, and MDSC expansion, improving survival in murine models.	Preclinical	[132,133]
	Targeting MDSCs: Functional Inhibition	- Metformin reduced CD39/CD73 expression and suppressed MDSC activity. - RA190 reversed MDSC-mediated suppression and restored CD8^+^ activity. - Dabigatran reduced MDSCs and enhanced cisplatin efficacy.	Preclinical	[134,135,136]
	Targeting MDSCs: Recruitment Blockade	- CXCR2/CXCR4 inhibition impaired trafficking. - PGE2/COX-2 inhibition reduced CXCR4 and limited MDSC accumulation.	Preclinical	[113,114]
	Targeting MDSCs: Differentiation Induction	Blocking IL-10 signalling reprogrammed MDSCs, boosted T-cell activity, and prolonged survival.	Preclinical	[137]
DCs	Dendritic Cell Vaccine (DCVAC/OvCa)	- Sequential administration after chemotherapy improved PFS (HR 0.39). - DCVAC + carboplatin/gemcitabine improved OS in relapsed OC.	Clinical (NCT02107937) (NCT02107950)	[138,139]

**Table 3:** Summary of studies on LMR/MLR as prognostic markers in ovarian cancer.

Study Design [Ref.]	Sample Size	Prognostic Markers Studied	Primary Outcomes	LMR/MLR Cutoff Value(s)	OS Hazard Ratio (95% CI)	PFS Hazard Ratio (95% CI)	Marker Type	Survival Impact	Disease Stage Association	Clinical Significance
Meta-analysis [20]	3346 patients from 12 studies	LMR	OS, PFS	Ranged from 1.85 to 4.35	1.85 (1.50–2.28) for high vs. low LMR	1.70 (1.49–1.94) for high vs. low LMR	Prognostic	High LMR is favourable. Stronger association in patients of <55 years.	Higher pretreatment LMR is associated with more favourable outcomes among all stages and subtypes of EOC/OC patients, and stronger associations for younger patients than older patients.	LMR is a simple, cost-effective prognostic biomarker. Future prospective trials are needed.
Meta-analysis [148]	2259 patients from 8 studies	LMR	OS, PFS, Clinicopathological features	Ranged from 1.85 to 4.2	1.92 (1.58–2.34) for low vs. high LMR	1.70 (1.54–1.88) for low vs. high LMR	Prognostic, correlative	Low LMR indicates a poor prognosis.	Low LMR is significantly associated with advanced FIGO stage (III–IV), higher tumour grade (G2/G3), lymph node metastasis, and malignant ascites.	Pre-treatment LMR is a potential marker of poor outcome and is correlated with aggressive tumour characteristics.
Meta-analysis [158]	2343 patients from 7 studies	LMR/MLR	OS, PFS, Clinicopathological parameters	Ranged from 2.22 to 4.35	1.81 (1.38–2.37) for low vs. high LMR	1.65 (1.46–1.85) for low vs. high LMR	Prognostic, correlative	Low LMR is associated with unfavourable survival.	Low LMR is significantly associated with advanced FIGO stage, lymph node metastasis, larger residual tumour, and higher CA-125 levels.	LMR could serve as a promising prognostic biomarker, particularly in China.
Meta-analysis [159]	2809 patients from 9 studies	LMR	OS, PFS	Ranged from 1.85 to 4.35	1.71 (1.40–2.09) for low vs. high LMR	1.68 (1.49–1.88) for low vs. high LMR	Prognostic	Lower LMR is associated with poorer OS and PFS.	Association is significant in both early and advanced stage disease groups.	LMR is a cheap and readily accessible prognostic tool. May assist in research on therapies modulating host immune response.
Retrospective Cohort [160]	214	LMR, CA-125, COLC (LMR + CA-125)	OS, PFS	LMR: 3.8	Low LMR: HR = 0.459 (0.306–0.688) (protective effect of high LMR)	Low LMR: HR = 0.494 (0.329–0.742) (protective effect of high LMR)	Prognostic	Low LMR and high CA-125 are independent predictors of poor OS and PFS.	Not specified	Combining LMR with CA-125 (COLC) improves prognostic specificity for mortality compared to either marker alone.
Retrospective Study [161]	92 patients with advanced OC	LMR in blood (bLMR), LMR in malignant fluid (mLMR)	PFS	bLMR: 2.80; mLMR: 2.41	Not studied	Low bLMR and low mLMR were independently associated with poor PFS.	Prognostic	Low values in both blood and malignant body fluids (ascites, pleural effusion) are associated with a poor prognosis.	Study limited to advanced stage (III–IV) OC	This is the first study validating the clinical value of LMR in malignant body fluids. A combined score (bmLMR) is a better predictor of recurrence.
Retrospective Cohort with PSM [145]	368 (matched to 111 vs. 111)	LMR	OS	LMR: 4.65	Low LMR: HR = 1.49 (*p* = 0.041) (increased risk for low LMR)	Not studied	Prognostic	Low preoperative LMR is an independent factor for poor OS after primary surgery.	Low LMR is significantly correlated with advanced FIGO stage, presence of ascites, and poor differentiation.	Low LMR had a noticeable negative effect in younger patients (<65) and those with aggressive tumours. Completing chemotherapy is crucial for low-LMR patients.
Retrospective Cohort [162]	146 (61 PDS, 85 IDS)	NLR, MLR, PLR	OS, PFS	MLR: 0.4	Not significant	Not significant	Prognostic (in different surgical settings)	In this study of Caucasian patients, MLR was not a significant prognostic factor in either the PDS or IDS groups.	Study limited to advanced stage (III–IV) OC	The timing of surgery may modulate the prognostic impact of inflammatory markers. High NLR and PLR were prognostic in the PDS group, but not the IDS group.

Abbreviations: AUC: Area Under the Curve; CA-125: cancer antigen 125; CI: confidence interval; COLC: combination of LMR and CA-125; EOC: epithelial ovarian cancer; FIGO: International Federation of Gynaecology and Obstetrics; HR: hazard ratio; IDS: Interval Debulking Surgery; LMR: lymphocyte-to-monocyte ratio; MLR: monocyte-to-lymphocyte ratio; NLR: neutrophil-to-lymphocyte ratio; OC: ovarian cancer; OS: overall survival; PDS: Primary Debulking Surgery; PFS: progression-free survival; PLR: platelet-to-lymphocyte ratio; PSM: propensity score matching; SIRI: Systemic Immune-Inflammation Index.

## Data Availability

No new data were created or analyzed in this study. Data sharing is not applicable to this article.

## Abbreviations

The following abbreviations are used in this manuscript:

AKT	Protein kinase B
ALDH+	Aldehyde dehydrogenase-positive
APCs	Antigen-presenting cells
ARG1	Arginase-1
C/EBPα	CCAAT/enhancer-binding protein alpha
C5	Complement component C5
CA-125	Cancer antigen 125
CAR-M	Chimeric antigen receptor macrophage
cDCs	Conventional dendritic cells
CMOP	Common monocyte progenitor
CSF-1	Colony stimulating factor 1
CSF-1R	Colony stimulating factor 1 receptor
DCs	Dendritic cells
EMT	Epithelial-mesenchymal transition
EOC	Epithelial ovarian cancer
ERK	Extracellular signal-regulated kinase
EVs	Extracellular vesicles
FIGO	International Federation of Gynaecology and Obstetrics
GM-CSF	Granulocyte-macrophage colony-stimulating factor
HE4	Human epididymis protein 4
HR	Hazard ratio
IDO	Indoleamine 2,3-dioxygenase
IFN-α	Interferon-alpha
IFN-γ	Interferon-gamma
IL	Interleukin
iNOS	Inducible nitric oxide synthase
IRF	Interferon regulatory factor
JAK	Janus kinase
KLF4	Krueppel-like factor 4
LMR	Lymphocyte-to-monocyte ratio
LPS	Lipopolysaccharide
MAPK	Mitogen-activated protein kinase
mDCs	Myeloid dendritic cells
MDSCs	Myeloid-derived suppressor cells
MHC	Major histocompatibility complex
MLR	Monocyte-to-lymphocyte ratio
MMCs	Myeloid mononuclear cells
M-MDSCs	Monocytic MDSCs
MMPs	Matrix metalloproteinases
NF-κb	Nuclear Factor kappa-light-chain-enhancer of activated B cells
NK	Natural killer
OC	Ovarian Cancer
OS	Overall survival
PD-1	Programmed death-1
pDCs	Plasmacytoid dendritic cells
PDGF	Platelet-derived growth factor
PD-L1	Programmed death-ligand 1
PFS	Progression-free survival
PGE_2_	Prostaglandin E_2_
PI3K	Phosphoinositide 3-kinase
PMN-/G-MDSCs	Polymorphonuclear or granulocytic MDSCs
ROC	Receiver operating characteristic
ROS	Reactive oxygen species
STAT	Signal Transducer and Activator of Transcription
TAMs	Tumour-associated macrophages
TGF-β	Transforming growth factor beta
TME	Tumour microenvironment
TNF-α	Tumour necrosis factor-alpha
Tregs	Regulatory T cells
VEGF	Vascular endothelial growth factor
